# Circulating tumor cells in newly diagnosed inflammatory breast cancer

**DOI:** 10.1186/s13058-014-0507-6

**Published:** 2015-01-09

**Authors:** Michal Mego, Antonio Giordano, Ugo De Giorgi, Hiroko Masuda, Limin Hsu, Mario Giuliano, Tamer M Fouad, Shaheenah Dawood, Naoto T Ueno, Vicente Valero, Eleni Andreopoulou, Ricardo H Alvarez, Wendy A Woodward, Gabriel N Hortobagyi, Massimo Cristofanilli, James M Reuben

**Affiliations:** Department of Hematopathology, The University of Texas MD Anderson Cancer Center, 1515 Holcombe Blvd, Houston, TX 77030 USA; Breast Medical Oncology, The University of Texas MD Anderson Cancer Center, Houston, TX USA; Radiation Oncology, The University of Texas MD Anderson Cancer Center, Houston, TX USA; Morgan Welch Inflammatory Breast Cancer Research Program and Clinic, The University of Texas MD Anderson Cancer Center, Houston, TX USA; Department of Medical Oncology, Comenius University, School of Medicine, Bratislava, Slovakia; Medical Oncology, Istituto Scientifico Romagnolo per lo Studio e la Cura dei Tumori (IRST) – IRCCS, Meldola, FC Italy; Department of Clinical Medicine and Surgery, University Federico II, Naples, Italy; Medical Oncology Department, Dubai Hospital, Dubai, UAE; Present affiliation: Breast Center, Thomas Jefferson University-Kimmel Cancer Center, Philadelphia, PA USA

## Abstract

**Introduction:**

Circulating tumor cells (CTCs) are an independent prognostic factor for progression-free survival (PFS) and overall survival (OS) in patients with metastatic breast cancer. Inflammatory breast cancer (IBC) is one of the most aggressive forms of breast cancer. The prognostic value of a CTC count in newly diagnosed IBC has not been established. The aim of this study was to assess the prognostic value of a baseline CTC count in patients with newly diagnosed IBC.

**Methods:**

This retrospective study included 147 patients with newly diagnosed IBC (77 with locally advanced and 70 with metastatic IBC) treated with neoadjuvant therapy or first-line chemotherapy during the period from January 2004 through December 2012 at The University of Texas MD Anderson Cancer Center. CTCs were detected and enumerated by using the CellSearch system before patients were started with chemotherapy.

**Results:**

The proportion of patients with ≥1 CTC was lower among patients with stage III than among patients with metastatic IBC (54.5% versus 84.3%; *P* = 0.0002); the proportion of patients with ≥5 CTCs was also lower for stage III than for metastatic IBC (19.5% versus 47.1%; *P* = 0.0004). Patients with fewer than five CTCs had significantly better progression-free survival (PFS) (hazard ratio (HR) = 0.60; *P* = 0.02) and overall survival (HR = 0.59; *P* = 0.03) than patients with five or more CTCs. Among patients with stage III IBC, there was a nonsignificant difference in PFS (HR = 0.66; 95% confidence interval (CI), 0.31 to 1.39; *P* = 0.29) and OS (HR = 0.54; 95% CI, 0.24 to 1.26; *P* = 0.48) in patients with no CTCs compared with patients with one or more CTCs. In multivariate analysis, CTC was prognostic for PFS and OS independent of clinical stage.

**Conclusions:**

CTCs can be detected in a large proportion of patients with newly diagnosed IBC and are a strong predictor of worse prognosis in patients with newly diagnosed IBC.

## Introduction

Inflammatory breast cancer (IBC) is one of the most aggressive forms of primary breast cancer, and the incidence of IBC is increasing [1]. The prognosis of patients with IBC remains poor: the 10-year disease-free survival rate is only 20% to 25%, despite a multimodality treatment approach [2-7]. These reports suggest that current treatment modalities are inadequate and underscore the need for better understanding of this disease.

IBC is associated with special clinical and biological features and a distinctive pattern of recurrence with high incidence of visceral metastases (central nervous system, lung, and liver) as first site of relapse [3-7]. It is characterized by a high proliferation rate, frequent hormone-receptor negativity, HER2 overexpression, high grade, and increased tumor angiogenesis [7-11]. Studies of several molecular factors in IBC suggest frequent epidermal growth factor receptor overexpression and high expression of p53, MUC1, RhoC, E-cadherin, and transcription factors associated with a stem cell phenotype [12-17].

In patients with metastatic breast cancer, circulating tumor cells (CTCs) are an independent predictor of progression-free survival (PFS) and overall survival (OS) [18]. In patients with metastatic disease, superior survival was observed among patients with fewer than five CTCs per 7.5 ml of peripheral blood regardless of histologic subtype, hormone receptor and HER2/neu status, sites of first metastasis, or whether the patient had recurrent or *de novo* metastatic disease [18-21]. The prognostic value of a CTC count was found to be superior to that of tumor burden as measured by Swenerton score or by serum tumor markers, ascribing a peculiar biological value to CTCs. These observations also raised the possibility that CTCs might represent a population of tumorigenic cancer cells with stem cell properties that might play an important role in tumor dissemination [22,23].

Previously, we showed that among patients with metastatic breast cancer (MBC) treated with first or subsequent lines of chemotherapy, patients with metastatic IBC had lower CTC counts than did patients with non-inflammatory metastatic breast cancer (non-IBC) [24]. We also showed, for patients with metastatic IBC, that differences in OS between patients with fewer than five CTCs and others with five or more CTCs were not statistically significant; hence, the prognostic value of a CTC count in patients with pretreated metastatic IBC is limited [24]. The prognostic value of a CTC count in newly diagnosed IBC has not been established.

In the present study, we investigated the prognostic value of a baseline CTC count and the relation between a baseline CTC count and primary tumor characteristics in patients with newly diagnosed IBC. Recent data suggest that statins might have anticancer effect in IBC, and their use was associated with prolonged PFS in primary IBC [25]. Therefore, we performed exploratory analysis to evaluate the relation between exposure to statin before diagnosis of IBC and CTC count.

## Methods

### Study patients

**Figure 1:**
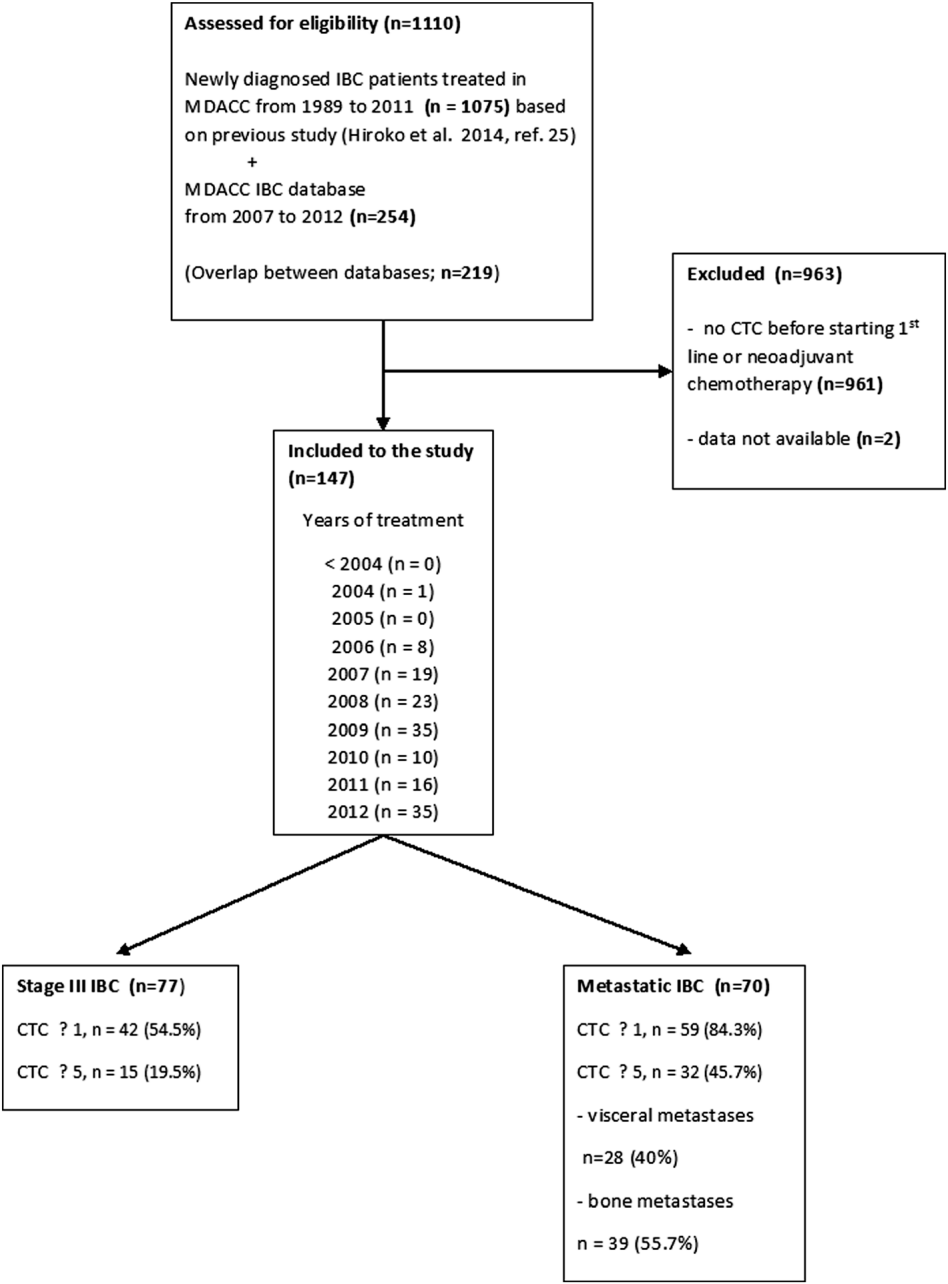
**Patients’ flow.**

This retrospective study was conducted under Institutional Review Board (IRB)-approved protocol DR10-0227 by using the Clinic Station, the MD Anderson Cancer Center (MDACC) electronic medical record database. Patients were identified by using two databases; database of newly diagnosed IBC patients treated in MD Anderson Cancer Center between 1989 and 2011, as described in the previous study [3], and the MDACC IBC database with available data from 2007 to 2012 (Figure 1). A population of consecutive stage III IBC and metastatic IBC patients with CTCs measurement before starting neoadjuvant or first-line treatment, was eligible. Only treatment-naïve patients with newly diagnosed disease, starting treatment with neoadjuvant or first-line chemotherapy, were included in this study. Patients underwent systemic therapy, as appropriate for their malignancies, irrespective of CTCs. Patients with concurrent malignancy other than nonmelanoma skin cancer in the previous 5 years were excluded.

All patients underwent pretreatment diagnostic biopsy. The diagnosis of IBC was based on clinical signs such as diffuse erythema, *peau d'orange*, tenderness, induration, and warmth [26,27]. The presence of dermal lymphatic emboli in the diagnostic pathology report was not mandatory for the pathological diagnosis of IBC. Clinical stage at diagnosis of primary disease was coded according to the criteria set forth in the sixth edition of the American Joint Committee on Cancer’s *AJCC Cancer Staging Manual* [28].

In all patients, data regarding age, menopausal status, tumor histologic subtype, hormone-receptor status, HER2 amplification status, type and number of sites of metastases, delivery of systemic therapy, and outcome (progression, survival, pathological complete remission) were recorded and compared with the presence and number of CTCs. Because statins might have an antitumor effect in IBC, we also recorded statin use before the diagnosis of IBC [25]. Lipophilic statins (simvastatin, fluvastatin, and lovastatin) were classified as L-statins, and weakly lipophilic-to-hydrophilic statins (atorvastatin, pravastatin, and rosuvastatin) were classified as H-statins, as described previously [25].

The retrospective study was approved by the Institutional Review Board of the University of Texas, MD Anderson Cancer Center, and a waiver of consent form was granted.

### Detection of CTCs in peripheral blood

The CellSearch system (Veridex Corporation, Warren, NJ, USA) was used to detect and enumerate CTCs in 7.5 ml of whole peripheral blood. Samples were subjected to enrichment of epithelial cells with anti-EpCAM-coated ferrous particles. CTCs were defined as nucleated cells (DAPI+) expressing cytoplasmic cytokeratins 8, 18, or 19 and lacking surface expression of the common leukocyte antigen (CD45) [18]. Specimens were stored at room temperature and processed for detection of CTCs by using CellSearch within 1 day of phlebotomy.

### Statistical analysis

Patient characteristics were tabulated. Baseline CTC count was defined as the earliest CTC measurement obtained before the start of a new line of therapy. We dichotomized baseline CTC counts in two different ways: as <1 or ≥1 and as <5 or ≥5. The cut-off at 1 CTC was chosen because it has been investigated in other settings, such as primary breast cancer, including locally advanced breast cancer [29,30]. The cut-off at 5 CTCs was established as prognostic for PFS and OS in patients with metastatic breast cancer in a previous study [18].

In an exploratory analysis, we correlated baseline CTC counts with PFS and OS. Median follow-up period was calculated as a median observation time among all patients and among those still alive at the time of their last follow-up. PFS was calculated from the date of baseline CTC enumeration to the date of progression or death or the date of last adequate follow-up. OS was calculated from the date of baseline CTC enumeration to the date of death or last follow-up. PFS and OS were estimated by using the Kaplan-Meier product-limit method and compared between groups by using the log-rank test. Univariate analyses were performed with either χ^2^ or Fisher Exact test, as appropriate. A multivariate Cox proportional hazards model for PFS and OS was used to assess differences in outcome on the basis of baseline CTC counts, hormone-receptor status (positive for either or negative for both), HER-2 status (overexpressed or negative), stage (stage III versus metastatic IBC), site of metastasis (visceral versus non-visceral), and number of metastatic sites. Visceral metastases were defined here as lung, liver, adrenal gland, brain, kidney, pancreas, and/or peritoneal involvement with or without ascites and/or pleural effusions. Nonvisceral metastases were defined as involvement of any of the following sites without visceral metastases: breast, lymph nodes, chest wall, bone, skin, and/or abdomen. Step-wise regression techniques were used to build multivariate models by using a significance level of 0.10 to remain in the model. Wilcoxon matched-pairs signed-ranks test was used to compare baseline CTC counts with CTC counts at the time of progression, and Mann–Whitney *U* test was used to compare CTC counts between patients with stage III IBC and metastatic IBC. All statistical tests were two-sided, and *P* values <0.05 were considered statistically significant.

## Results

### Patient characteristics

A total of 147 patients with newly diagnosed IBC that matched the study eligibility criteria was included in this analysis. Of these 147 patients, 77 had locally advanced (stage IIIB and IIIC) and 70 had metastatic IBC. Thirteen patients (8.7%) with newly diagnosed metastatic IBC (mIBC) in this analysis were also included in a previous report [24]. The median age of the subjects was 54 years (range, 23 to 82 years). One hundred forty patients (95.2%) had invasive ductal carcinoma. Patients’ characteristics and the prevalence of baseline CTCs are shown in Table 1.

**Table 1:** **Patient characteristics and prevalence of circulating tumor cells at baseline (** ***n*** **= 147)**

	**Stage III IBC**						**Metastatic IBC**					
**Characteristic**	***N***	**%**	**≥1 CTC** ^**a**^	***%***	***≥*** **5 CTC** ^**a**^	***%***	***N***	**%**	***≥*** **1 CTC** ^**a**^	***%***	***≥*** **5 CTC** ^**a**^	***%***
All patients	77	100.0	42	54.5	15	19.5	70	100.0	59	84.3	32	45.7
Histology												
Infiltrative ductal carcinoma	74	96.1	40	54.1	15	20.3	66	94.3	55	83.3	31	47.0
Other histology	3	3.9	2	66.7	0	0.00	4	5.7	4	100.0	1	25.0
*P* value				1.00		0.61				0.61		0.62
ER/PR status												
Positive for either	43	55.8	19	44.2	5	11.6	40	57.1	33	82.5	20	50.0
Negative for both	34	44.2	23	67.6	10	29.4	30	42.9	26	86.7	12	40.0
*P* value				0.06		0.08				0.74		0.47
HER-2/neu status												
Overexpressed	26	33.8	17	65.4	7	26.9	19	27.1	13	68.4	9	47.4
Negative	51	66.2	25	49.0	8	15.7	51	72.9	46	90.2	23	45.1
*P* value				0.23		0.36				0.04		1.00
Grade												
High grade	55	71.4	32	58.2	11	20.0	53	75.7	43	81.1	24	45.3
Intermediate/low grade	22	28.6	10	45.5	4	18.2	15	21.4	14	93.3	8	53.3
Unknown	-	-	-	-	-	-	2	2.9	2	-	0	-
*P* value				0.31		1.00				0.43		0.77
ER/PR and HER2/neu status												
Triple receptor negative	19	24.7	12	63.2	5	26.3	19	27.1	17	89.5	7	36.8
Not-triple receptor negative	58	75.3	30	51.7	10	17.2	51	72.9	42	82.4	25	49.0
*P* value				0.44		0.50				0.71		0.43
Sites of metastases												
Non-visceral	-	-	-	-	-	-	42	60.0	34	81.0	18	42.9
Visceral	-	-	-	-	-	-	28	40.0	25	89.3	14	50.0
*P* value										0.50		0.63
Bone metastasis												
Present	-	-	-	-	-	-	39	55.7	32	82.1	19	48.7
Absent	-	-	-	-	-	-	31	44.3	27	87.1	13	41.9
*P* value										0.70		0.63
Number of metastases												
1	-	-	-	-	-	-	29	41.4	22	75.9	13	44.8
≥2	-	-	-	-	-	-	41	58.6	37	90.2	19	46.3
*P* value										0.20		1.00
Statin use												
No-statins	62	80.5	39	62.9	14	22.6	60	85.7	51	85.0	30	50.0
L-statins^b^	7	9.1	1	14.3	1	14.3	5	7.1	5	100.0	1	20.0
H-statins^b^	8	10.4	2	25.0	0	0.0	5	7.1	3	60.0	1	20.0
*P* value				0.01		0.30				0.20		0.21
Menopausal status												
Premenopausal	27	35.1	19	70.4	9	33.3	25	35.7	21	84.0	13	52.0
Postmenopausal	50	64.9	23	46.0	6	12.0	45	64.3	38	84.4	19	42.2
*P* value				0.06		0.03				1.00		0.46
Lymphovascular tumor emboli												
Present	36	46.8	20	55.6	9	25.0	42	60.0	37	88.1	17	40.5
Absent	40	51.9	21	52.5	6	15.0	24	34.3	18	75.0	12	50.0
Unknown	1	1.3	1	-	0	-	4	5.7	4	-	3	-
*P* value				0.64		0.39				0.19		0.61

ER, estrogen receptor; PR, progesterone receptor.

^a^Per 7.5 ml of whole peripheral blood.

^b^Lipophilic statins were classified as L-statins, and weakly lipophilic to hydrophilic statins were classified as H-statins.

### Role of CTCs in IBC

Median baseline CTC count among the 147 patients assessed for the presence of CTCs was 2 (range, 0 to 249) per 7.5 ml of peripheral blood (PB). Among the 147 patients, a subset of 101 patients (68.7%) had at least one CTC, whereas 48 patients (32.7%) had at least five CTCs.

The median CTC counts in patients with stage III and mIBC were 1 (range, 0 to 179) and 3 (range, 0 to 249) (*P* < 0.0001), respectively. The proportion of patients with one or more CTCs was lower in patients with stage III than in patients with mIBC (54.5% versus 84.3%; *P* = 0.0002); the proportion of patients with five or more CTCs was also lower for stage III than for mIBC (19.5% versus 47.1%; *P* = 0.0004). The proportion of patients with one or more and five or more CTCs was higher in premenopausal than in postmenopausal women in stage III IBC but not in mIBC patients (Table 1), and there was a trend to detect CTCs more often in patients with than in patients without lymphovascular tumor emboli (74.4% versus 59.4%; *P* = 0.07).

**Figure 2:**
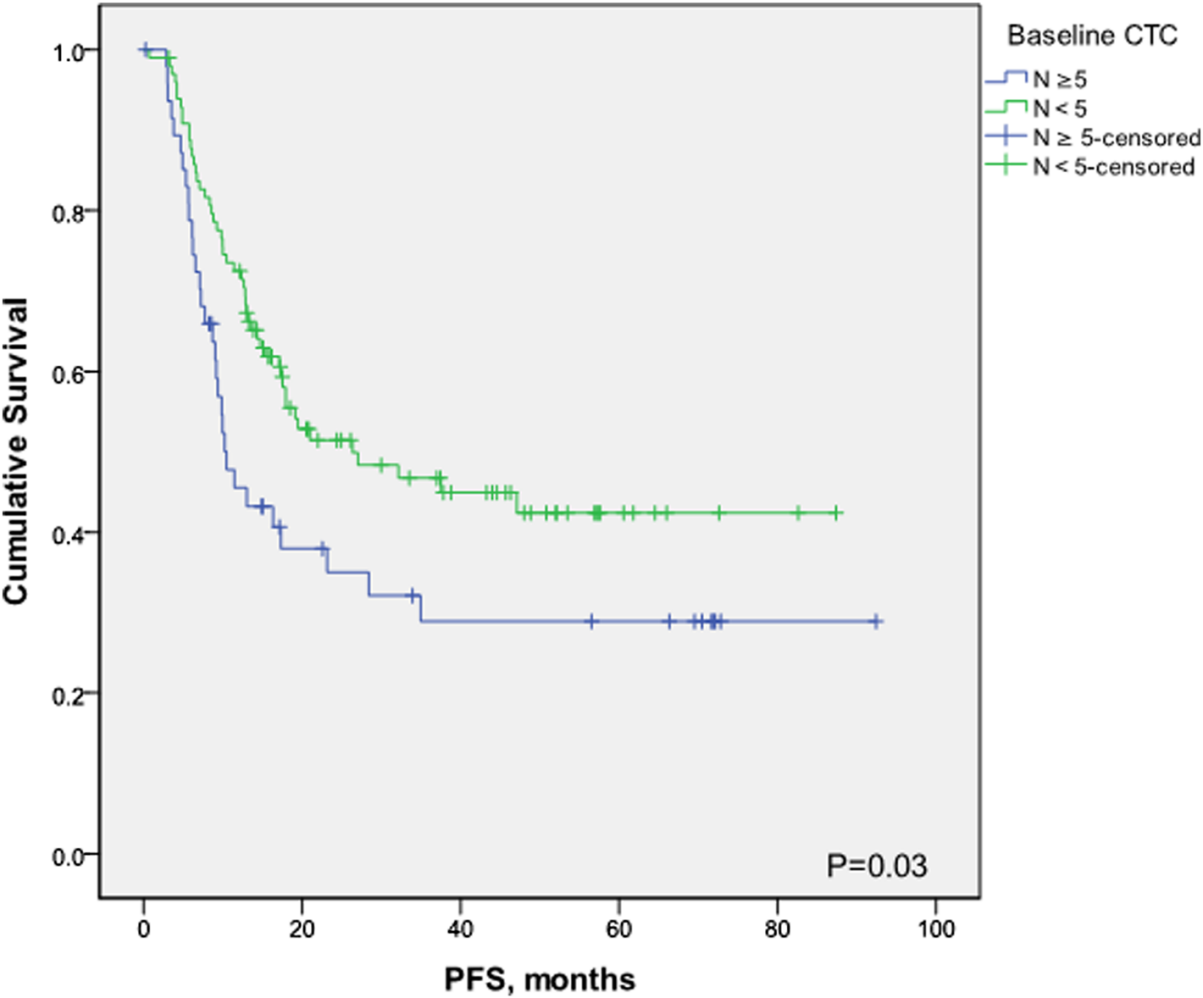
**Kaplan-Meier estimates of probabilities of progression-free survival, according to baseline circulating tumor cell count in patients with newly diagnosed inflammatory breast cancer.**

**Figure 3:**
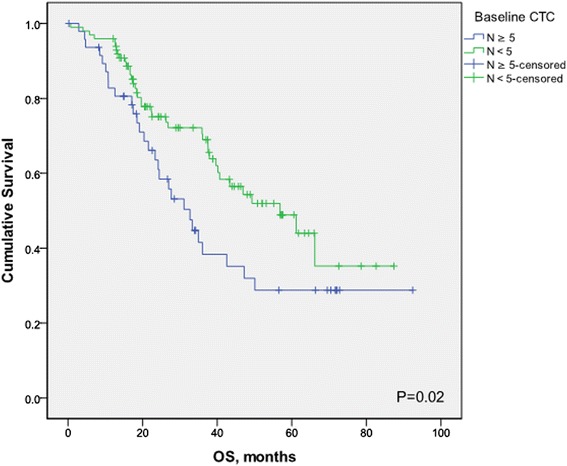
**Kaplan-Meier estimates of probabilities of overall survival, according to baseline circulating tumor cells count in patients with newly diagnosed inflammatory breast cancer.**

At a median follow-up time of 26.3 months (range, 1.0 to 92.4 months), 81 patients (55.1%) had experienced disease progression, and 66 patients (44.9%) had died. Median follow-up of patients still alive was 35.6 (range, 8.2 to 92.4 months). Patients with fewer than five CTCs had a significantly better PFS than patients with five or more CTCs (median PFS, 26.4 versus 10.5 months; hazard ratio [HR] = 0.60; 95% CI, 0.37 to 0.98; *P* = 0.02) (Figure 2). Furthermore, patients with fewer than five CTCs had a significantly better OS than patients with five or more CTCs (median OS, 56.9 versus 32.7 months; HR = 0.59; 95% CI, 0.35 to 1.00; *P* = 0.03) (Figure 3). Similarly, with a cut-off of one CTC, patients with fewer than one CTCs had a significantly better PFS (HR = 0.42; 95% CI, 0.27 to0.66; *P* = 0.001) and OS (HR = 0.35; 95% CI, 0.21 to 0.58; *P* = 0.001) compared with patients with one or more 1CTCs.

**Figure 4:**
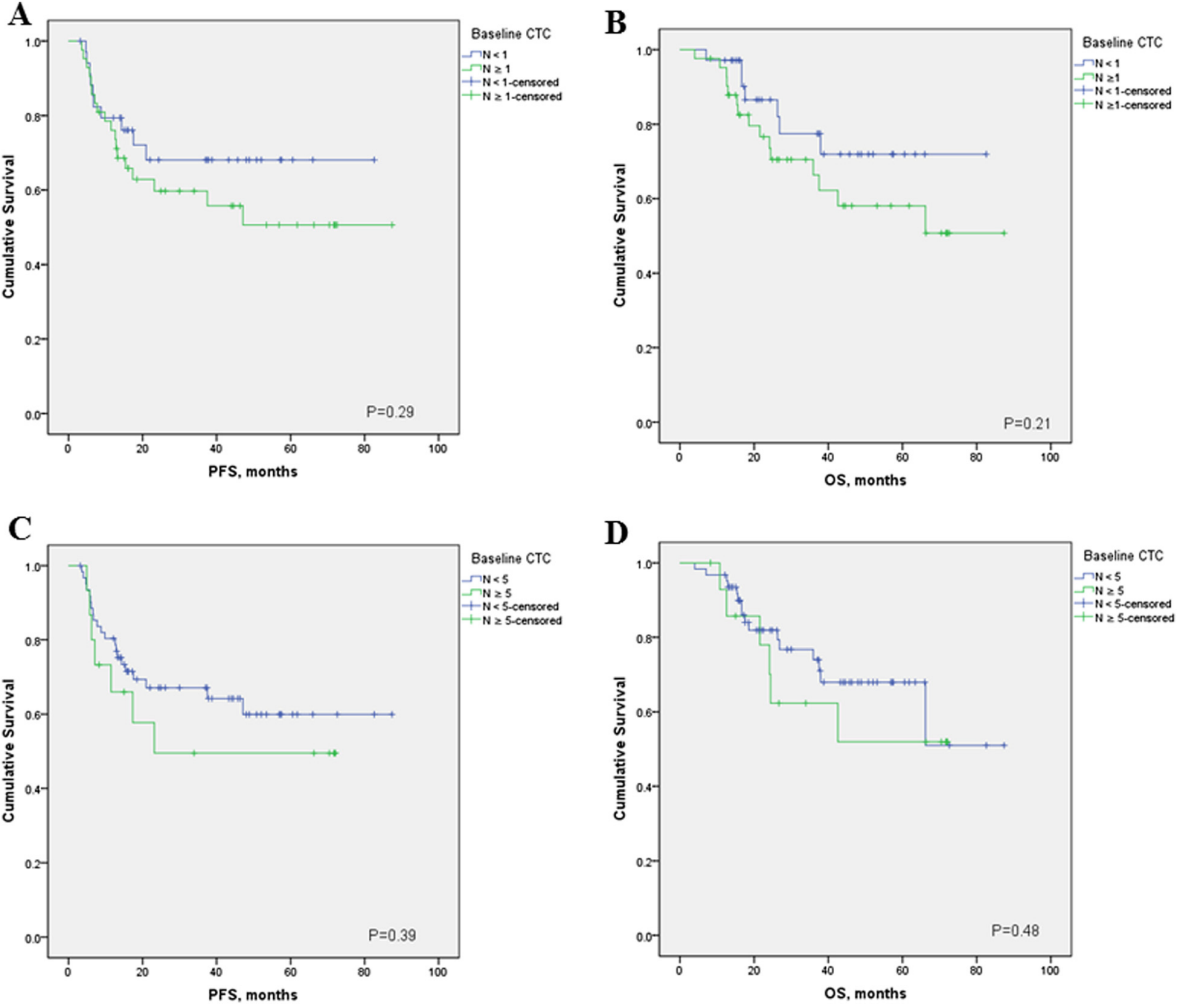
**Kaplan-Meier estimates of probabilities of progression-free survival (A, cut-off 1 CTC; C, cut-off 5 CTCs), and overall survival (B, cut-off 1 CTC; C, cut-off 5 CTCs cut-off 1 CTC; D, cut-off 5 CTCs) according to baseline circulating tumor cell count in patients with newly diagnosed stage III inflammatory breast cancer.**

**Figure 5:**
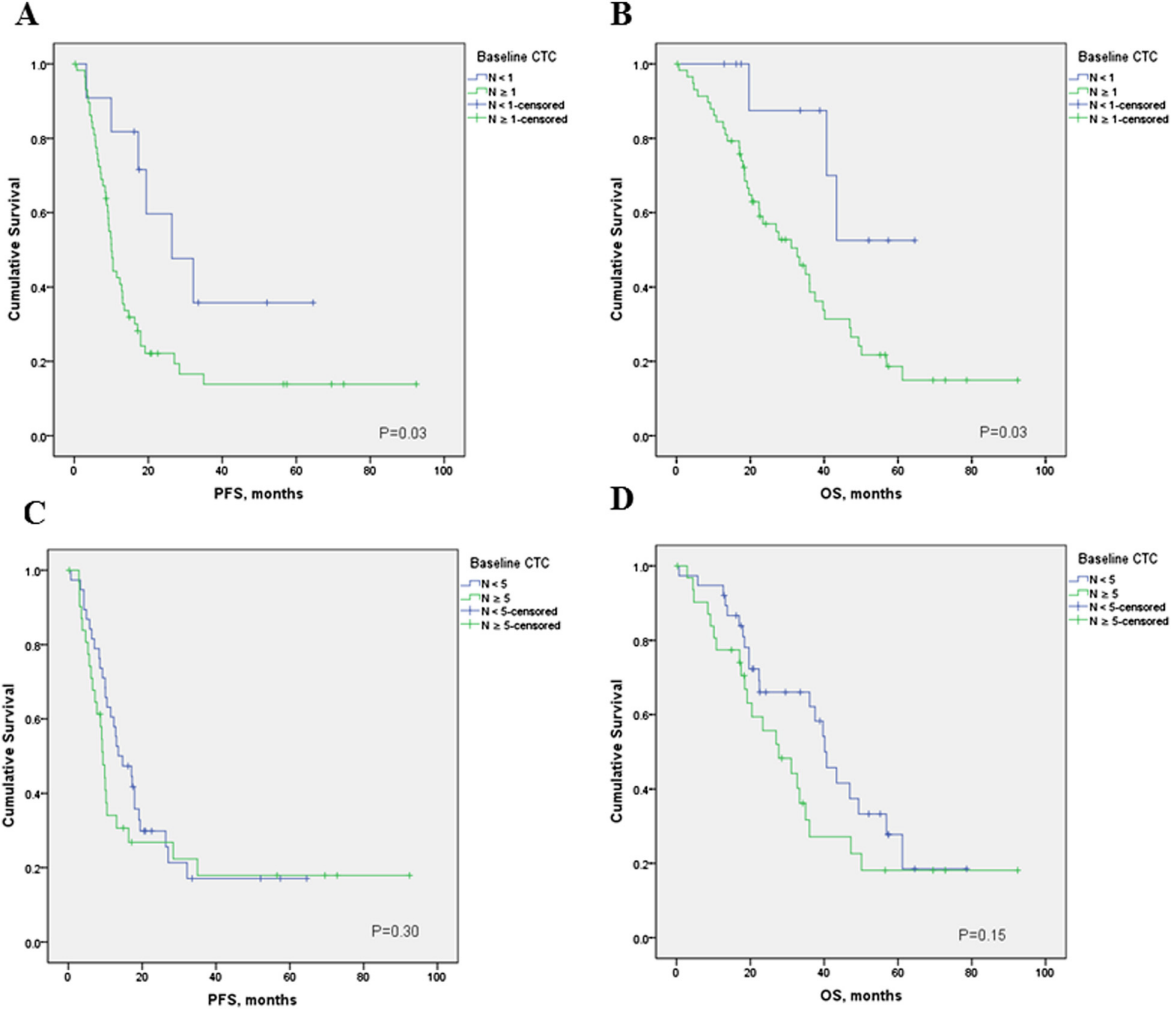
**Kaplan-Meier estimates of probabilities of progression-free survival (A, cut-off 1 CTC; C, cut-off 5 CTCs), and overall survival (B, cut-off 1 CTC; C, cut-off 5 CTCs cut-off 1 CTC; D, cut-off 5 CTCs) according to baseline circulating tumor cell count in patients with newly diagnosed metastatic IBC.**

Tables 2 and 3 and Figures 4 and 5 summarize the Kaplan-Meier PFS and OS estimates by CTC count and patient tumor characteristics for stage III IBC and metastatic IBC patients. CTC prognostic value was observed only in metastatic IBC patients, with a threshold of one CTC (Table 3). In multivariate analysis baseline CTCs, HER2 status and stage of disease were independent prognostic factors for PFS, whereas baseline CTCs, hormone-receptor status, HER2 status, and visceral metastases were independent prognostic factors for OS (Table 4). Same results were obtained by using a cut-off of one or more CTCs (data not shown).

**Table 2:** **Kaplan-Meier progression-free survival (PFS) and overall survival estimates stratified by CTC level and patient and tumor characteristics in patients with newly diagnosed stage III inflammatory breast cancer**

	**Progression-free survival**				**Overall survival**			
**Variable**	**Hazard ratio**	**Lower 95% CI**	**Upper 95% CI**	***P*** **value**	**Hazard ratio**	**Lower 95% CI**	**Upper 95% CI**	***P*** **value**
All patients								
<1 CTC versus ≥1 CTC	0.66	0.31	1.39	0.29	0.57	0.25	1.31	0.21
<5 CTC versus ≥5 CTC	0.69	0.26	1.78	0.39	0.72	0.26	2.00	0.48
ER/PR positive for either								
<1 CTC versus ≥1 CTC	0.59	0.23	1.53	0.28	0.59	0.18	1.94	0.40
<5 CTC versus ≥5 CTC	0.33	0.06	1.70	0.04	0.33	0.05	2.18	0.08
ER/PR negative for both								
<1 CTC versus ≥1 CTC	0.76	0.22	2.69	0.69	1.14	0.55	2.39	0.70
<5 CTC versus ≥5 CTC	1.14	0.31	4.14	0.85	1.05	0.28	3.91	0.94
HER-2/neu positive								
<1 CTC versus ≥1 CTC	0.64	0.15	2.77	0.58	0.37	0.07	1.97	0.34
<5 CTC versus ≥5 CTC	1.25	0.27	5.71	0.79	0.97	0.18	5.33	0.97
HER-2/neu negative								
<1 CTC versus ≥1 CTC	0.62	0.26	1.49	0.29	0.51	0.19	1.37	0.18
<5 CTC versus ≥5 CTC	0.42	0.11	1.59	0.08	0.51	0.13	2.05	0.23
Triple negative								
<1 CTC versus ≥1 CTC	0.40	0.11	1.51	0.24	0.31	0.08	1.14	0.11
<5 CTC versus ≥5 CTC	0.56	0.11	2.71	0.39	0.60	0.13	2.82	0.45
High grade								
<1 CTC versus ≥1 CTC	0.65	0.28	1.47	0.31	0.42	0.17	1.06	0.09
<5 CTC versus ≥5 CTC	0.56	0.19	1.69	0.22	0.44	0.13	1.46	0.09
Low/intermediate grade								
<1 CTC versus ≥1 CTC	0.87	0.15	5.08	0.87	1.34	0.18	9.70	0.76
<5 CTC versus ≥5 CTC	1.44	0.20	10.50	0.74	NA^a^	NA^a^	NA^a^	0.19

ER, estrogen receptor; NR, not reached; PR, progesterone receptor; NA, not applicable.

^a^No events in patients with five or more CTCs.

**Table 3:** **Kaplan-Meier progression-free survival (PFS) and overall survival (OS) estimates stratified by CTC level and patient and tumor characteristics in patients with newly diagnosed metastatic IBC**

	**Progression-free survival**				**Overall survival**			
**Variable**	**Hazard ratio**	**Lower 95% CI**	**Upper 95% CI**	***P*** **value**	**Hazard ratio**	**Lower 95% CI**	**Upper 95% CI**	***P*** **value**
All patients								
<1 CTC vs. ≥ 1 CTC	0.40	0.21	0.75	0.03	0.29	0.14	0.62	0.03
<5 CTC vs. ≥ 5 CTC	0.76	0.43	1.31	0.30	0.65	0.35	1.19	0.15
ER/PR positive for either								
<1 CTC vs. ≥ 1 CTC	0.30	0.13	0.67	0.03	0.14	0.05	0.37	0.02
<5 CTC vs. ≥ 5 CTC	0.57	0.27	1.20	0.13	0.54	0.24	1.22	0.14
ER/PR negative for both								
<1 CTC vs. ≥ 1 CTC	0.69	0.24	2.00	0.54	0.64	0.19	2.19	0.54
<5 CTC vs. ≥ 5 CTC	0.97	0.41	2.27	0.94	0.67	0.26	1.73	0.37
HER-2/neu positive								
<1 CTC vs. ≥ 1 CTC	0.22	0.05	0.90	0.11	NA ^a^	NA ^a^	NA ^a^	0.11
<5 CTC vs. ≥ 5 CTC	0.82	0.20	3.32	0.78	0.48	0.10	2.36	0.38
HER-2/neu negative								
<1 CTC vs. ≥ 1 CTC	0.75	0.32	1.72	0.53	0.52	0.21	1.29	0.26
<5 CTC vs. ≥ 5 CTC	0.66	0.36	1.22	0.16	0.58	0.30	1.14	0.09
Triple negative								
<1 CTC vs. ≥ 1 CTC	0.92	0.22	3.79	0.91	0.79	0.20	3.04	0.74
<5 CTC vs. ≥ 5 CTC	0.34	0.10	1.14	0.01	0.35	0.10	1.28	0.02
High Grade								
<1 CTC vs. ≥ 1 CTC	0.38	0.19	0.77	0.03	0.23	0.10	0.54	0.03
<5 CTC vs. ≥ 5 CTC	0.61	0.31	1.18	0.11	0.54	0.26	1.12	0.08
Low/intermediate grade								
<1 CTC vs. ≥ 1 CTC	0.75	0.12	4.54	0.77	1.05	0.13	8.45	0.96
<5 CTC vs. ≥ 5 CTC	1.30	0.44	3.87	0.61	0.87	0.28	2.71	0.81
Visceral metastases								
<1 CTC vs. ≥ 1 CTC	0.13	0.05	0.32	0.01	0.00	0.00	0.00	0.03
<5 CTC vs. ≥ 5 CTC	0.66	0.30	1.45	0.28	0.49	0.21	1.16	0.07
Non-visceral metastases								
<1 CTC vs. ≥ 1 CTC	0.66	0.28	1.56	0.39	0.62	0.22	1.77	0.44
<5 CTC vs. ≥ 5 CTC	0.87	0.40	1.88	0.71	0.78	0.33	1.83	0.55
Bone metastasis								
<1 CTC vs. ≥ 1 CTC	0.29	0.13	0.66	0.03	0.18	0.06	0.50	0.06
<5 CTC vs. ≥ 5 CTC	0.67	0.31	1.43	0.28	0.51	0.22	1.20	0.12
No-bone metastasis								
<1 CTC vs. ≥ 1 CTC	0.56	0.21	1.51	0.33	0.47	0.16	1.41	0.29
<5 CTC vs. ≥ 5 CTC	0.78	0.34	1.79	0.55	0.78	0.32	1.87	0.56

ER, estrogen receptor; NR, not reached; PR, progesterone receptor; NA, not applicable.

^a^No events in patients with fewer than one CTCs.

**Table 4:** **Multivariate analysis**

**Variable**	**PFS**		**OS**	
	**HR (95% CI)**	***P*** **value**	**HR (95% CI)**	***P*** **value**
**ER/PR status**	0.630	0.051	0.468	0.004
Positive for either versus negative for both	(0.400 - 1.002)		(0.279 - 0.785)	
**HER2 status**	0.309	<0.001	0.268	<0.001
Overexpressed versus negative	(0.173 - 0.554)		(0.138 - 0.521)	
**Sites of metastases**	1.536	0.143	2.319	0.002
Visceral versus nonvisceral	(0.865 - 2.729)		(1.364 - 3.942)	
**Stage**	2.939	<0.001	1.681	0.131
mIBC versus stage III	(1.818 – 4.750)		(0.857 – 3.298)	
**Baseline CTCs count**	1.621	0.044	1.995	0.008
≥5 versus <5	(1.014 - 2.591)		(1.202 - 3.309)	

### Relation of Statin and CTCs in IBC

We identified 25 patients (17%) that used statins before the diagnosis of IBC (Table 1). Interestingly, an inverse association was noted between the use of statins and the presence of CTCs. The proportion of patients with ≥1 CTCs was lower in patients taking statins than in those not taking statins (44.0% versus 73.8%; *P* = 0.005); the proportion of patients with ≥5 CTCs was also lower in patients taking statins (12.0% versus 36.9%; *P* = 0.02). This effect was more striking for patients using H-statins than for patients using L-statins.

### Role of CTCs in Primary IBC

In nonmetastatic breast cancer patients, a cut-off at one CTC is established based on previous trials [29-31], therefore, we analyzed the prognostic value of CTCs by using this cut-off in stage III IBC patients as well. Among patients with stage III IBC, non-significant differences occurred in PFS (HR = 0.66; 95% CI, 0.31 to 1.39; *P* = 0.29) and OS (HR = 0.54; 95% CI, 0.24 to 1.26; *P* = 0.48) in patients with no CTCs compared with those of patients with one or more CTCs (Figure 4). In exploratory analysis, we evaluated the prognostic value of CTCs by using a cut-off at 5. Similarly to these data, patients with fewer than 5 CTCs had a nonsignificantly better PFS (HR = 0.69; 95% CI, 0.26 to 1.78; *P* = 0.38) and OS (HR = 0.75; 95% CI, 0.27 to 2.06; *P* = 0.53) than patients with five or more CTCs.

Of the 77 patients with stage III IBC, 15 (19.2%) achieved a pathologic complete response (pCR) after treatment with neoadjuvant chemotherapy; however, no correlation was found between baseline CTCs count and pCR. Table 5 shows association between CTCs and pCR in stage III IBC patients.

**Table 5:** **Association between baseline CTCs and pathologic complete remission (pCR) in stage III IBC**

	**non-pCR (%)**	**pCR (%)**	***P*** **value**
CTC < 1	30 (85.7)	5 (14.3)	0.39
CTC ≥ 1	32 (76.2)	10 (23.8)	
CTC < 5	53 (85.5)	9 (14.5)	0.06
CTC ≥ 5	9 (60.0)	6 (40.0)	

## Discussion

To our knowledge, this is the first study to assess the prognostic value of CTCs in patients with newly diagnosed IBC. This study indicates that baseline CTCs, as enumerated by the CellSearch technology, are prognostic for PFS and OS in patients with newly diagnosed mIBC. The proportion of patients with a baseline CTC count of ≥1 in stage III and in mIBC was 54.5% and 84.3%, respectively; that is much higher than the proportions previously reported in patients with non-IBC, even for those with metastatic disease. In contrast, the proportion of patients with five or more CTCs in metastatic IBC was 45.7%, within the range previously observed in patients with metastatic breast cancer [18-20]. We also confirmed the findings of previous reports that tumors in patients with IBC frequently were hormone-receptor negative, were of high grade, and overexpressed HER2 [7-9,11].

We also found that the proportion of patients with CTCs was lower in patients with nonmetastatic IBC than in those with metastatic IBC (54.5% versus 84.3%; *P* = 0.0002). The previously reported prevalence of CTCs among patients with early primary non-IBC is lower (range, 21% to 38%) [29,30,32-36] than that of CTCs among patients with stage III IBC in this study (55.5%). In a phase II study of HER2-positive primary inflammatory breast cancer patients, the prevalences of patients with one or more CTCs and five or more CTCs were 35% and 13%, respectively, and lower compared with our study, which includes HER2-negative patients as well [36]; unfortunately, we are not aware of any data on CTC prevalence in patients with newly diagnosed locally advanced non-IBC, a more appropriate group for comparison with our patients with stage III IBC.

In our study, we did not observe a correlation between pathologic complete remission and patients’ outcome, and similar to previous reports, we observed a lack of correlation between baseline CTC count and pathologic complete remission [32,33,36].

We observed that IBC patients with CTC counts of less than five had significantly better outcomes than IBC patients with CTC counts of five or more. In a previous study of patients with metastatic IBC, we found the CTC count to be of limited prognostic value; differences in OS between patients with CTC counts of less than five and CTC counts of five or more were not significant [24]. In that study, we observed a lower prevalence of CTCs and fewer CTCs in patients with metastatic IBC than in patients with metastatic non-IBC [24]. However, the vast majority of those patients had received neoadjuvant or first-line therapy. In our current study, we observed that CTC counts at the time of disease progression after chemotherapy were lower than CTC counts at baseline, and that the proportions of patients with CTC counts of five or more or with one or more CTCs were significantly lower at the time of disease progression than at baseline, which is consistent with our earlier report [24].

Emerging data suggest that statins, in addition to their known antiinflammatory effects, might also have an antiproliferative effect on breast cancer cells; however, available evidence on breast cancer risk is conflicting [37,38]. Statins are usually well tolerated, but their administration is associated with some important side effects, including myositis, rhabdomyolysis, hepatotoxicity, and diarrhea. A recent cohort study showed that use of weakly lipophilic to hydrophilic statins (H-statins) is associated with significantly improved PFS compared with no statin use in patients with IBC [25]. In our study, we observed that patients who took statins before the diagnosis of IBC had significantly lower baseline CTC counts than patients not taking statins. Consistent with previous observations, patients who used H-statins had lower baseline CTC counts compared with patients without statins. These data, even though hypothesis generating, further add credence to the earlier observation that statins might have an anticancer effect, especially in IBC, and warrant additional study.

## Conclusion

In conclusion, this retrospective study suggests a prognostic value of CTCs in patients with newly diagnosed IBC. We observed that patients with nonmetastatic IBC had a lower CTC prevalence and lower CTC counts at progression than did patients with metastatic IBC. Further research should focus on characterization of CTCs in IBC patients and the implications for treatment decisions.

### Ethics approval

Institutional Review Board, The University of Texas, MD Anderson Cancer Center, protocol DR10-0227.

## Abbreviations

CD45+: CD45 positive
CTC: circulating tumor cell
DAPI: 4′,6-diamidino-2-phenylindole
IBC: inflammatory breast cancer
H-statins: hydrophilic statins
L-statins: lipophilic statins
MBC: metastatic breast cancer
mIBC: metastatic IBC
MDACC: The MD Anderson Cancer Centre
non-IBC: non-inflammatory metastatic breast cancer
OS: overall survival
pCR: pathologic complete response
PFS: progression-free survival
